# Effects of propolis, royal jelly, honey and bee pollen on growth performance and immune system of Japanese quails

**Published:** 2016-03-15

**Authors:** Sakine Babaei, Shaeban Rahimi, Mohammad Amir Karimi Torshizi, Golamhosein Tahmasebi, Seyed Naser Khaleghi Miran

**Affiliations:** 1 *Department of Poultry Science, Faculty of Agriculture, Tarbiat Modares University, Tehran, Iran; *; 2 *Department of Honeybee and Silkworm Research, Animal Science Research Institute, Karaj, Iran.*

**Keywords:** Cell mediated immunity, Feed conversion, Honey bees products, Humoral immunity

## Abstract

Effect of ethanolic extract of propolis, royal jelly, honey and bee pollen in comparison with virginiamycin (as growth promoter antibiotic) as regards the performance and immune system of Japanese quail were assessed. We used 256 mixed-sex quail chicks in a completely randomized design by eight treatments, including control, ethanolic extract of propolis 1000 (PE1) and 5000 (PE2) mg kg^-1^, pollen powder 1000 (PO1) and 5000 (PO2) mg kg^-1^, royal jelly 100 mg kg^-1^ (RJ), honey 22 g L^-1^ of drinking water (H) and virginiamycin 150 mg kg^-1^ (V), and four replicates of eight birds in each replication for 42 days. There was significant differences for weight gain (WG), feed intakes (FI) and feed conversion ratio (FCR) between experimental groups. In addition, WG in V treatment (18.82%), H and PO2 treatments (16.87%) and RJ treatment (12.00%) were significantly higher than to control group (*p* < 0.01). Significantly higher values of FCR were recorded in control group while group PE2 exhibited a significant decrease for 1 to 42 day (*p* < 0.05). The results of effect of bee products on antibodies titer showed an increase in the Newcastle disease (ND) titer when compared with control and virginiamycin groups (*p* < 0.01). There was significant difference in antibody production against avian influenza (AI) and sheep red blood cells (*p* < 0.01). Significant differences were observed in heterophils to lymphocytes ratio among PE1, PE2, PO1 and PO2 with V and control groups (*p* < 0.01).

## Introduction

Antibiotics have been added to poultry feed to improve the growth performance, to stabilize intestinal microflora and to prevent infection by specific pathogenic micro-organisms. In poultry production, it is very important to improve immunity so as to prevent infectious disease. Minimizing immunosuppression and its impact is also an important strategy for success in the poultry industry. Utilization of immunostimulants is one solution to improve the immunity of animals and to decrease their susceptibility to infectious diseases.^1^ Antibiotic growth promoters for poultry diets have been banned for use in European Union and pressure from consumer groups and major poultry buyers has threatened their removal from diets in the US. Therefore, studies on alternate products that can result in promotion of growth, improved feed utilization, and maintenance of gut health are taking place.^2^ Propolis (bee glue) is a natural resinous hive product collected by bees from plants, particularly from flowers and leaf buds. Propolis contains a variety of chemical compounds such as poly-phenols (flavonoid aglycones, phenolic acids and their esters, phenolic aldehydes, alcohols, and ketones), terpenoids, steroids, amino acids and inorganic compounds.^3^^,^^4^ Many biological properties, including anti-bacterial, antifungal, antiviral, antioxidant, hepatoprotective and immuno-stimulating activities of propolis have been reported.^4^^,^^10^ Modern herbalists recommend propolis for human use in medicine because of its antibacterial, anti-fungal, antiviral, hepatoprotective and anti-inflammatory properties to increase the body’s natural resistance to infections and to treat gastro-duodenal ulcers. ^11^

In some countries the bee pollen has been recognized as food and medicine.^12^ Bee pollen contains at least 22 amino acids, 18 vitamins, 25 minerals, 59 trace elements, 11 enzymes or coenzymes, 14 fatty acids, 11 carbohydrates and approximately 25.00% protein. Bee pollen is extremely rich in carotenes, which are metabolic precursors of vitamin A. It is also high in vitamin B complex and vitamins C, D, E and lecithin. Bee pollen contains over 50.00% more protein than beef, yet its fat content is very low. Bee pollen contains digestive enzymes from the bees. Pollen may be used to improve the immune response, to reduce the effect of radiation and retards aging because of its antioxidant and flavonoid contents.^13^ Honey has been used since ancient times as part of traditional medicine. Several functions such as antibacterial, antioxidant, antitumour, anti-inflamatory, antibrowning, and antiviral have been reported.^14^ Royal jelly contains considerable amounts of proteins, amino acids including eight essential amino acids,^15^ hormone rich substance (testosterone) has been identified in extremely small quantities in royal jelly about 12 mg g^-1^ fresh weight. Royal jelly also contains vitamins: A, C, D, E, minerals are in descending order: K, Ca, Na, Zn, Fe, Cu, Mn, enzymes and antibiotic components. It also has an abundance of nucleic acid DNA and RNA.^16^ Royal jelly has been determined to exhibit a variety of pharmacological activities including antitumor, antimicrobial, antioxidant activity, vasodilative and hypotensive activities, as well as growth stimulating and infection preventing, anti-hypercholesterolemic and anti-inflammatory activities.^17^

The aim of this study was to determine the effect of royal jelly, honey, bee pollen and alcoholic extracted propolis on performance and immune system of Japanese quails.

## Materials and Methods

**Birds and husbandry.** The experiment enrolled 256 of one-day-old quail chicks. They were weighed individually and randomly allocated to eight dietary treatments so that each experimental unit had equal average weight and distribution of eight birds. Experimental groups consisting of: a control diet (not supplemented), ethanolic extract of propolis 1000 mg kg^-1^ (PE1) and 5000 mg kg^-1 ^(PE2), pollen powder 1000 mg kg^-1^ (PO1) and 5000 (PO2) mg kg^-1^, royal jelly 100 (RJ) mg kg^-1^, honey 22 g L^-1^ (H) and virginiamycin 150 mg kg^-1^ (V). The quail chicks were kept in cages (40 × 32 cm^2^). Experimental diets were fed from 1-42 days of age. Feed and water were available *ad libitum*. The diets were formulated based on soybean-corn, to meet or exceed NRC requirements. ^18^ The experimental groups were replaced with sand in diets during grower periods. Ingredient composition and nutrient analysis is described in Table 1.

**Table 1:** Composition of the experimental diet

***Ingredients ***	**Percentage in diet**
**Corn**	50.70
**Soybean meal**	42.52
**Vegetable oil**	2.00
**Di-Calcium phosphate**	0.72
**Calcium carbonate**	1.25
**Salt**	0.33
**Vitamin premix** 1	0.25
**Mineral premix** 2	0.25
**DL-methionine**	0.13
**L-Threonine**	0.11
**Sand**	1.74
***Nutrient composition***	
**Metabolizable Energy (Kcal kg** ^-1^ **)**	2800
**Crude protein (%)**	23.00
**Calcium (%)**	0.77
**Available phosphorus (%)**	0.29
**Sodium (%)**	0.15
**Methionine (%)**	0.48
**Methionine + Cysteine (%)**	0.85
**Lysine (%)**	1.28
**Threonine (%)**	0.98

1 Provides per kg of diet, Vitamin A: 3125 μg, Vitamin D_3_: 75 μg, α-tocopherol acetate: 50 mg, Vitamin K3: 5 mg, Vitamin B1: 3 mg, Vitamin B_2_: 6 mg, Vitamin B_6_: 5 mg, Vitamin B_12_: 0.003 mg, Pan-tothenic acid: 10 mg, Niacin: 50 mg, Folic acid: 1 mg, Biotin: 0.1 mg.

2 Provides per kg of diet, Cu: 5 mg, I: 2 mg, Co: 0.5 mg, Se: 0.15 mg, Mn: 90 mg, Fe: 50 mg, Zn: 70 mg.

**Sample preparation and extraction. **Propolis, pollen, royal jelly and honey were collected of honey bee, Animal Science Research Institute, Karaj, Iran. Propolis was kept desiccated in the dark until processing. A 30.00% propolis tincture was prepared by adding 600 g propolis to 1400 mL 70.00% ethanol (w/v). This was mixed and kept in a glass container, shaken twice daily, filtered after one week and kept at 4 ˚C until it was used.^19^

**Performance.** Body weight (BW) and feed intake (FI) per replication were recorded on days 21 and 42, and these values were used to calculate weight gains (WG), FI and feed conversion (FCR) of the quail chicks for the periods between days 0-21, 21-42, as well as for the overall experiment.

**1-chloro-2, 4-dinitrobenzene (DNCB) test.** On 42 days of age, 1 mg mL^-1 ^DNCB solution (Merck, Darmstadt, Germany) was spread and maintained over a 2 cm^2^ area of featherless skin (0.05 mL) on the right side of the two birds per cage. Similar position on the left side of the bird treated by the solvent alone (acetone: olive oil, 4:1 v/v) to correct the solvent effect. The skin swelling was calculated as the difference between the thickness of the skin before and after DNCB treatment was measured using a digital caliper.^20^

**Mitogen injection** **.** At six weeks of age, two chicks from each cage were injected with mitogen phytohemagglutinin (PHA-M; Gibco, Grand Island, USA) into the left wing web, intradermally. Each chick received 50 μL of a suspension of 50 μg PHA-M in 50 μL phosphate buffered saline.^21^ A digital caliper (Mitutoyo, Kawasaki, Japan) was used to measure the wing web thickness before injection (as a control measurement), and at 24 hr after injection to obtain the wing web swelling in response to the mitogen.^22^

**Humoral immune response. **Vaccination against Newcastle disease (ND) were performed on day seven using eye drop (live B1 strain; Razi Institute, Karaj, Iran) and AI H9N2 subcutaneous injection on day 21 (Newcastle and avian influenza killed vaccines; Razi Institute) to all chicks. As a booster, the second live ND vaccine (La Sota strain) was performed via drinking water at day 23. The immune response was assessed by hemagglutination inhibition (HI) test.^23^ The HI titers against both vaccines were determined on serum samples of the same birds at day 42. The sheep red blood cells (SRBC) were collected and washed three times in phosphate buffer saline (PBS). The packed cells were brought to 5.00% in sterile PBS solution. Chicks were injected into breast muscle with SRBC (0.1 mL per chick) followed by a booster injection at days 28 and 35, respectively. Blood samples were drawn on day 42. Serum was stored at – 20 ˚C until tested. The antibody levels against SRBC were measured by HI test.^24^^,^^25^

**Determination of body and lymphoid organ weights** **.** The birds slaughtered on day 42 were eviscerated and bursa and spleen were removed and weighed. The immune organ index was expressed as the weight of the organ as a percentage of the organ to the total body weight.

**Evaluation of the** **heterophil to lymphocyte ratio.** Blood smears for differential leukocyte counts were prepared using the standard two-slide wedge procedure. The samples were air dried, fixed in methanol and stained with wright quick stain. Smears were examined to obtain counts of lymphocytes and granulocytes per 100 leukocytes. Obtained cell counts were used for calculation of the relative proportion of heterophils to lymphocytes (H/L ratio).^26^

**Statistical analysis. **The data were analyzed by generalized linear model (GLM) procedure for completely randomized experimental design with seven treatments and four replicates using SAS (Version 8.2; SAS Institute, Cary, USA) and the means were compared by Tukey’s test. A *p* value less than 0.05 was considered as statistically significant.

## Results

**Performance. **The effects of the treatments on quail performance are presented in Table 2. There was significant difference in weight gain from day 1 to 21, between H with PE2, PO2, V and C (*p* < 0.05). The highest weight gain was related to H. There was significant difference in weight gain from day 21 to 42, between V and other experimental groups, except for H, RJ and PO2 (*p* < 0.01). There was significant difference between experimental groups PE1, PE2, PO1 and control with V in weight gain from day 1 to 42 (*p* < 0.01).

**Table 2:** Effect of honey bee products on daily live weight gain, daily feed intake and feed conversion ratio of Japanese quails

**Treatment**	**Weight gain (g per bird day** ^-1^ **)**				**Feed intakes (g per bird day** ^-1^ **)**				**Feed conversion ratio**		
	**1-21**	**21-42**	**1-42**		**1-21**	**21-42**	**1-42**		**1-21**	**21-42**	**1-42**
**PE1**	1.49abc	5.55^d^	3.52^cd^		3.47^bc^	14.69^bc^	9.08^b^		2.33^ab^	2.65^ab^	2.49^ab^
**PE2**	1.42^bc^	5.75^cd^	3.58^cd^		3.42^bc^	11.61^d^	7.51^c^		2.41^ab^	2.02^d^	2.21^d^
**PO1**	1.52^ab^	6.02bcd	3.77bcd		3.93^a^	14.62^bc^	9.27^ab^		2.57^a^	2.43abc	2.50^ab^
**PO2**	1.32^c^	6.98^a^	4.15^ab^		3.20^c^	14.47^c^	8.84^b^		2.42^ab^	2.07^cd^	2.24^cd^
**RJ**	1.48abc	6.37abc	3.92abc		3.65^ab^	15.68abc	9.67^ab^		2.47^a^	2.46^ab^	2.46abc
**V**	1.39^bc^	7.11^a^	4.25^a^		3.54^bc^	16.47^a^	10.00^a^		2.53^a^	2.32bcd	2.42abcd
**H**	1.60^a^	6.71^ab^	4.15^ab^		3.50^bc^	15.86^ab^	9.68^ab^		2.19^b^	2.41^bc^	2.30bcd
**C**	1.37^bc^	5.53^d^	3.45^d^		3.44^bc^	15.24abc	9.34^ab^		2.51^a^	2.77^a^	2.64^a^
**SEM**	0.0185	0.1176	0.0593		0.0418	0.2619	0.1367		0.0258	0.0482	0.0281
***p*** **-value**	0.0125	0.0001	0.0070		0.0060	0.0001	0. 0015		0.0247	0.0001	0.0131

PE1: 1000 and PE2: 5000 mg kg^-1^ ethanolic extract of propolis, PO1: 1000 and PO2: 5000 mg kg^-1 ^pollen powder, RJ: 100 mg kg^-1^ royal jelly, H: honey 22 g L^-1^, C: control and V: virginiamycin 150 mg kg^-1^. SEM: Standard error of mean.

abcd different superscripts indicate statistically differences in each column (*p *< 0.05).

In addition, WG in V treatment (18.82%), H and PO2 treatments (16.87%) and RJ treatment (12.00%) were significantly higher than to control group (*p* < 0.01). There were significant differences for FI and FCR between treatment groups at 21 and 42 days of age. In addition, PO1 and V supplementation increased FI of quail chicks from 1 to 21 and 21 to 42 days respectively (*p* < 0.01). There was significant difference in FI from 1 to 42 day between treatments, but these values was significantly decreased in group PE2 in comparison with other groups (*p* < 0.01). The FCR was significantly affected by H, PE1, PE2 and PO2 from 1 to 21 day (*p* <0.01). There was significant difference in FCR from 21 to 42 day, between PE2, PO2, V and H with control group (*p* < 0.01). Significantly higher values of FCR were recorded in control group while group PE2 exhibited a significant decrease for 1 to 42 day (*p *< 0.05). 

**Immunity.** The differences in antibodies titer of Japanese quails in different groups were summarized in Table 3. Serum antibody titer is a valid indicator of the humoral immunity in quails and plays an important role in the host’s defense against infections. The results of effect of bee products on antibodies titer showed an increase in the ND titer when compared with control and virginiamycin groups. However, PE1 and PO2 numerically produced the highest antibody titers for ND compared with control and virginiamycin groups at the end of experiment (*p* < 0.01). There was significant difference in antibody production against AI (*p* < 0.01). The highest concentration related to H and PO2 for AI. There was significant difference for total anti-SRBC antibody titer (on 42 day). The highest antibody titer belonged to PO2 for SRBC. The effects of the treatments on cell-mediated immunity by response of skin to DNCB and wing web swelling by PHA are presented in Table 4. 

**Table 3:** Comparison between antibody titrations against New-castle (ND), avian influenza H9N2 (AI) viruses and total anti-sheep red blood cell (SRBC) and heterophil/lymphocyte (H/L) ratio of Japanese quails

**Treatment**	**ND (log** ^2^ ** HI)**	**AI (log** ^2^ ** HI)**	**SRBC**	**H/L**
**PE1**	6.00^a^	3.50^a^	1.50^b^	0.16^c^
**PE2**	3.60^b^	4.25^a^	1.75^ab^	0.15^c^
**PO1**	4.00^b^	3.25^ab^	1.75^ab^	0.12^d^
**PO2**	5.75^a^	4.50^a^	3.00^a^	0.15^cd^
**RJ**	3.75^b^	3.75^a^	2.50^ab^	0.19^ab^
**V**	3.50^b^	4.00^a^	1.50^b^	0.22^a^
**H**	4.75^ab^	4.50^a^	2.00^ab^	0.17^bc^
**C**	3.50^b^	2.00^b^	1.50^b^	0.21^a^
**SEM**	0.1936	0.1694	0.1265	0.008
***p*** **-value**	0.0001	0.0001	0.0063	0.0001

PE1: 1000 and PE2: 5000 mg kg^-1^ ethanolic extract of propolis, PO1: 1000 and PO2: 5000 mg kg^-1 ^pollen powder, RJ: 100 mg kg^-1^ royal jelly, H: honey 22 g L^-1^, C: control and V: virginiamycin 150 mg kg^-1^. SEM: Standard error of mean.

abcd Different superscripts indicate statistically differences in each column (*p *< 0.05).

There was a significant increase in skin thickness of H, PO1 and PO2 in comparison with control group for DNCB (*p *< 0.01). Skin thickness response to PHA was not affected by the experimental groups at day 42 (*p* > 0.05). But PE1, PO2 and H numerically increased the wing web response to PHA in comparison with the rest of experimental groups. Data of relative weights of bursa and spleen are summarized in Table 4. There was no significant difference in weight of bursa and spleen (*p* > 0.05). The highest increase in weight of bursa was observed in RJ group. PO1 group numerically increased the weight of spleen in comparison with other groups. Significant differences were observed in heterophil to lymphocyte ratio between PE1, PE2, PO1 and PO2 with V and control groups (*p* < 0.01) (Table 3). The lowest H/L ratios were obtained in birds fed diets containing PO1.

**Table 4:** Effect of experimental groups on relative lymphoid organ weights and cell-mediated immunity by response of skin to dinitrohlorebenze (DNCB) and wing web swelling by phytohemagglutinin (PHA

**Treatment**	**Increase in skin thickness (%) to PHA and DNCB**			**Relative lymphoid organ weights (g per 100 g body weight)**	
	**PHA**	**DNCB**		**Bursa**	**Spleen**
**PE1**	0.65	1.81^bc^		0.09	0.09
**PE2**	0.55	1.75^bc^		0.10	0.08
**PO1**	0.64	2.32^ab^		0.09	0.09
**PO2**	0.83	2.46^ab^		0.10	0.08
**RJ**	0.30	1.53^c^		0.14	0.09
**V**	0.94	2.26abc		0.12	0.08
**H**	0.76	2.67^a^		0.10	0.06
**C**	0.74	1.55^c^		0.11	0.07
**SEM**	0.0629	0.0884		0.0046	0.0032
***p*** **-value**	0.3282	0.0001		0.2307	0.1231

PE1: 1000 and PE2: 5000 mg kg^-1^ ethanolic extract of propolis, PO1: 1000 and PO2: 5000 mg kg^-1 ^pollen powder, RJ: 100 mg kg^-1^ royal jelly, H: honey 22 g L^-1^, C: control and V: virginiamycin 150 mg kg^-1^. SEM: Standard error of mean.

abcd different superscripts indicate statistically differences in each column (*p *< 0.05).

## Discussion

Results of this experiment indicated that addition of 5000 mg kg^-1^ propolis, pollen 5000 mg kg^-1^ and honey 22 g L^-1^ could be beneficial in improving quail chicks performance. Weight gain increased in V group (18.80%), H and PO2 groups (16.90%), RJ group (12.00%), PO1 group (9.30%), PE2 group (3.70%) and PE1 group (2.00%) compared to control group. A similar finding was reported that *Alternanthera* *brasiliana* and propolis extracts increased body weight gain from 14 to 21 days.^27^ Increased dietary PE2 and PO2 supplementation and 2.20% aqueous honey tended to improve feed efficiency. These findings are not in agreement with the results of some researchers who indicated that addition of propolis ethanolic extract to quail diets did not affect the feed efficiency.^28^ Also, it was reported that propolis supplementation at levels of 500 and 2000 mg kg^-1^ diet did not improve the performance of male broilers.^29^ In another study it was reported that addition of propolis powder at 0.5, 1.0 and 1.5 g kg^-1^ diet increased the growth parameters of quail chicks.^30^ Supplementation of propolis in broiler diets at a level of 500 mg kg^-1^ increased body weight gain by 20.00%.^31^ This improvement of WG could be due to the high content of flavonoids in propolis diets and increased FI compared to control group.^31^ Experimental work of some investigators showed that propolis supplementation to the ration of pullets improved FCR. FI and FCR significantly decreased by increasing levels of pollen and propolis supplementation (*p*> 0.05).^32^ When bee pollen extract were added (400 mg kg^-1^ and 800 mg kg^-1^) in feed mixtures for feeding broiler chickens, BW was increased in experimental groups compared to control group.^33^ This result confirmed the results of studies by Benkova,^34^ Angelovicova *et al*., ^35^ and Seven *et al*.^36^ who had added propolis to chickens feed. They had found that the BW in experimental groups were higher than control group. This has been attributed to its several enzymes which support the digestive system to increase the FCR. Besides, by giving a good flavor to food, increases the rate of FI.^33^

Royal jelly has lots of flavonoids, organic compounds and fatty acids. These components can improve growth performance of the quails resulting in increased BW, FI and lower FCR. The impact of active ingredients, especially flavonoids, on quails performance in stress conditions was reported previously.^37^

In conclusion, under the conditions investigated, honey bee products resulted in improvement of growth performance. Therefore, propolis, royal jelly, honey and pollen can be recommended as a growth promoter in quail production. Findings of the current study in respect to heterophil and lymphocyte counts showed that significant decrease in H/L ratios was detected in all treated groups with bee products in comparison with control and V groups, this results could be due to the proliferation of lymphocytes in the groups receiving bee products. These results are consistent with some studies which reported the effect of honey bee product on heterophil, lymphocyte, eosinophil, monocyte, basophil and H/L ratio. These effects could be explained by the stimulatory effects of bee products on immune functions and improved immunocompetence of the birds.^38^ The H/L ratio has been a reliable index for determining stress in poultry.^26^ The differential leukocyte count can be used to estimate the impact of dietary additives on the animal’s health.^39^ The inclusion of 1.00% to 4.00% of propolis extraction residue in broiler diets was not affected percentage of lymphocyte, heterophil, basophil, and eosinophil and the H/L ratio.^40^ Heterophil and lymphocyte number and their ratio were affected by *in ovo* injection of RJ increasing significantly the H number and H/L ratio, while decreasing significantly the L number compared with other groups.^41^ Addition of honey to drinking water of heat-stressed birds had no significant effect on heterophil, lymphocyte, eosinophil, monocyte, basophil and H/L ratio.^38^ Royal jelly injection in broiler chickens decreased the number of lymphocyte and increased the heterophil count as well as their ratio as compared to control group.^41^ It has been reported that the number of lymphocytes in chicken peripheral blood decreases and the number of heterophils increases in response to stressors and to increasing levels of corticosterone.^26^ There is also evidence that this ratio is influenced by diseases and infections or the stress hormones produced as a result of the infection.^42^ However, in this study, the H/L ratio was significantly decreased. 

The current findings of the effects of different levels of bee products and virginiamycin on humoral and cell-mediated immune response implied an increase in phagocytic activity and phagocytic index in all treated groups with the highest value observed in PE2 group. Similar effects were reported with medicinal plants. Broiler chicks fed honey had significantly higher antibody response as compared with control group. It is concluded that honey in drinking water may be considered as a new natural additive due to its micronutrients capable of enhancing and developing the immune system.^43^ Serum antibody titer is an indicator of humoral immunity. Results of this study also showed that the antibody titers in most of treatment groups were higher than that from control group, suggesting that they could promote humoral immunity. The total antibody response of bee products additives as supplement to quail chicks vaccinated with ND vaccine was evaluated. Quail chicks fed PE1 and PO2 had significantly higher antibody response as compared with virginiamycin and control groups. Previous research has found that the lowest antibody titers against ND were obtained in control group, with no pollen, indicating that PO improves the immune response to vaccines or challenges up to 28 days of age, but thereafter, antibody titers were similar in all treatments.^48^ Also, it was reported that there was no influence of dietary bee pollen inclusion on the humoral response of birds at 42 days of age.^48^ There was significant difference in antibody titers against AI and SRBC (*p* < 0.01). The highest concentration related to H and PO2 groups for AI and PO2 for SRBC. In the case of animal immunization with SRBC, in chickens there has been noticed a significant increase in antibodies production among those birds who were administered RJ^44^ and in rats and mice the serum levels of total proteins and immunoglobulins have significantly dropped and there has been an increase in plaque-forming splenocytes, in the weight of the inguinal lymph node and in the number of peripheral lymphocytes.^45^ Experimental work of some investigators showed that antibiotics had no effect on the antibody response to SRBC,^47^ whereas Brisbin *et al*. observed that chickens immunized and treated with either dose of virginiamycin (11 and 22 ppm) had an enhanced systemic antibody response to a soluble antigen, keyhole limpet hemocyanin (KLH), in their sera compared with immunized birds that did not receive virginiamycin^46^. The mechanism whereby virginiamycin enhances the immune responsiveness is not known. However, it is possible that the observed increase in anti-KLH-specific IgG and IgM is related to changes in the composition of the intestinal microbiota of antibiotic-treated birds.^46^ There was a significant increase in skin thickness of H, PO1 and PO2 compared to control group for DNCB (*p* < 0.01). The PHA was not affected by the experimental groups at day 42 (*p* > 0.05). But PE1, PO2 and H groups the wing web response to PHA was numerically increased in comparison with the rest of experimental groups. The relative weights of lymphoid organs were not affected in quails fed diets with the bee products (*p* > 0.05). In agreement our results, some researcher found that there was no significant differences in the relative weight of the spleen and bursa of Fabricius in broilers fed diet containing thyme compared with control groups.^48^ Excessive growth of these lymphoid organs may indicate an infection and more mortality in birds. Therefore, bee pollen could be used as a potential feed additive with prebiotic activity to the poultry diet. Positive effects of bee pollen on the chickens health is supported by the findings of some studies that reported an early development of thymus and bursa of Fabricius, then decrease in degeneration of the cloacal bursa and promotion of the splenic immune response, as well as an early development of the small intestine in broiler chicks.^47^^,^^48^ The RJ, H, PO, PE are natural bee products with a great potential for use in medicine. The use of honey bee products as a broiler immunostimulant for poultry farming purposes needs validation with further studies using different levels and other poultry species.^48^

Results of this study showed that the antibody titers in most of treatment groups at each time point were higher than that of control group, suggesting that they could promote humoral immunity. Previous studies have shown that flavonoids have an immunossupressor effect on the lymphoproliferative response.^43^ According to this study the micronutrient components of natural additive of honey and royal jelly added to drinking water of quails, ethanolic extract of propolis and pollen powder added to diet may improve performance and the development of the immune system of Japanese quails.

From our results, it could be concluded that H, PE2 and PO2 supplementation increases BW, lymphoid organ weight and antibody titer in Japanese quails. This may be due to the antimicrobial and immunostimulant activity of honey bee products. It opens perspective uses of honey, pollen and propolis as feed additive to improve poultry performance.
